# Identification of T-Cell Epitopes and Vaccine Development for African Swine Fever Virus

**DOI:** 10.3390/vaccines13090955

**Published:** 2025-09-07

**Authors:** Wanyi Ni, Hanchun Yang, Nianzhi Zhang

**Affiliations:** National Key Laboratory of Veterinary Public Health Security, Key Laboratory of Animal Epidemiology of the Ministry of Agriculture and Rural Affairs, College of Veterinary Medicine, China Agricultural University, Beijing 100193, China; niwanyi2001@163.com

**Keywords:** ASFV, vaccine, T-cell epitope, identification problems

## Abstract

African swine fever virus (ASFV) has inflicted severe devastation on the global pig industry, yet a globally approved vaccine remains unavailable. Given that cellular immunity is critical for ASFV prevention, the development of vaccines based on T-cell epitopes emerges as a promising strategy to control this virus. This review synthesizes the recent advancements and challenges in the research on ASFV T-cell epitopes, while offering insights into the potential impact of novel T-cell epitope-based vaccines. Notably, only a limited number of ASFV T-cell epitopes have been experimentally identified to date, covering fewer than 20 ASFV proteins. This bottleneck is attributed to challenges such as high swine leukocyte antigen (SLA) polymorphism, suboptimal accuracy of predicting tools, and complex experimental validation procedures. Although current studies on ASFV-specific T-cell immune responses and epitope identification are insufficient to meet vaccine development needs, continuous progress in T-cell immunology research in recent years has brought this goal closer to reality.

## 1. Present State of ASFV Vaccine Research

ASF is an extremely contagious viral hemorrhagic disease with significant fatalities in both farmed and wild pigs, severely affecting the economic advancement of the worldwide swine industry. A WOAH report published in November 2024 indicates that ASF has resulted in the loss of almost 1,794,000 animals globally since January 2022. As of now, over 80 nations have reported instances of ASF, which constitutes an enormous threat to food security and biodiversity worldwide (woah.org, access on 12 December 2024). Currently, the prevention and management of ASFV relies on rigorous import and export regulations and bio-safety disinfection protocols, with no viable vaccine or appropriate therapy available [1].

ASFV is the only member of the family *Asfarviridae*. It transmits efficiently among pigs, primarily via direct contact through the oronasal route or skin abrasions. Indirect transmission also contributes substantially to its spread: pigs are highly susceptible to infection through ingestion of contaminated substances (e.g., feed, water, etc.), or contact with contaminated environmental surfaces, fomites, and pork products [2]. ASFV is categorized as a nucleoplasmic large DNA virus (NCLDV), with a maximum diameter of 250 nm [3]. Its genome spans approximately 170–193 kbp and encodes over 160 proteins, including 68 structural proteins and more than 100 non-structural proteins [4]. ASFV particles possess a multilayered architecture, comprising an external lipid envelope, an icosahedral protein capsid, an inner lipid envelope, a protein core shell, and a nucleoid from the exterior inward [5,6,7]. The major capsid protein p72 comprises the majority of the outer capsid shell [3,7], and other minor capsid proteins establish a hexagonal network beneath the outer capsid shell [3,8,9]. The protein core shell is a distinct structural region of the virus, mostly composed of the polyprotein pp220 (p5, p14, p34, p37, p150) and the polyprotein pp62 (p8, p15, p35), both of which are susceptible to digestion by the protease pS273R [5,10]. Furthermore, the external lipid envelope (p12, pE402R) is related to the host cell receptor binding process; the inner lipid envelope (p17, p12, p22, pE183L, pE248R, pH108R, pE199L) is connected to the assembly of the capsid layer and the viral integration process; and the nucleoid layer comprises the viral genome encased by a dense protein layer (p10, pA104R) [7,11,12]. The intricate makeup and architecture of the virus particles are key factors in the current difficulties in developing an effective ASFV vaccine [6,7].

ASFV could interfere with the host immune system to suppress and evade the host immune response [13]. Various proteins in ASFV antagonize the host innate immune response via multiple pathways [7]. CD2v, A179L, and A224L evade the immune response by regulating macrophage apoptosis to facilitate viral infection [14,15,16]. ASFV proteins also inhibit the expression and secretion of cytokines. The MGF family inhibits Interleukin-1β (IL-1β) expression [17]; CD2v, C129R, EP364R, E120R, pA104R, DP96R, and the MGF family target and block the cGAS-STING and JAK-STAT cellular pathways to suppress type I interferon (IFN-I) expression and secretion [17,18,19,20,21,22,23,24]; additionally, pA238L has been shown to inhibit tumor necrosis factor-α (TNF-α) expression [25]. ASFV also antagonizes innate immunity by suppressing inflammation. pA238L and MGF360-12L block the NF-κB pathway to inhibit inflammation [18,26]; I226L, A151R, NP419L, QP383R, and the MGF family suppress inflammatory vesicle AIM2 activation [17,27]. Moreover, ASFV exhibits suppressive effects on adaptive immunity response. ASFV has been demonstrated to affect antigen presentation by down-regulating major histocompatibility complex class II (MHC-II) expression [28], inhibiting antigenic peptide processing, and blocking MHC-II binding to peptides [29]. In addition, EP153R protein may inhibit the transport of MHC-I [30], and A238L has been found to suppress the proliferation and activation of T cells [7]. The formidable immunosuppression ability of ASFV is another significant obstacle to vaccine development.

To date, no globally recognized, safe, and effective commercial ASF vaccine has been developed. From a safety perspective, inactivated vaccines and subunit vaccines represent the ideal selection. Research indicates that inactivated ASF vaccines fail to induce effective protective immunity [31,32]; the protective effect of these vaccines cannot be improved even with modern adjuvants (Polygen™ and Emulsigen-D^®^) [33]; high-dose inactivated vaccines, despite being safe, demonstrate poor immunogenicity [34]; and more intact virus particles obtained by inactivating ASFV with γ-radiation also failed to enhance its protective power [35]. In recent years, functions performed by numerous proteins in ASFV during the infection of host cells have been discovered [36,37]. Thus, subunit vaccines have become an important aspect of ASFV vaccine development during the last few years. However, the immunoprotection offered by the currently available ASFV subunit vaccines is inadequate. In experimental animals infected with ASFV after vaccination, the hosts still showed clinical symptoms and pathological characteristics [13,38,39]. For instance, inoculation with a mixture of baculovirus-expressed p30, p54, and p72 has shown ineffectiveness against the virulent ASFV isolate Pr4 [40]; likewise, immunization with mixtures of various vector antigens failed to improve the protective efficacy of subunit vaccines [38]. Given that inactivated vaccines and subunit vaccines consistently show inferior protective efficacy, the development of attenuated vaccines has emerged as a popular part of the current focus.

In comparison to inactivated vaccines and subunit vaccines, ASFV attenuated vaccines show substantial protective effects, making them a focal point for vaccine research. ASFV attenuated vaccines can be developed by three methods: natural attenuation, cell subculture, and gene knockout. Several natural attenuated vaccines have been created (e.g., Lv17/WB/Rie1 [41], NH/P68 [42], OURT88/3 [43]), but their efficacy fluctuates based on the administration route, dosage, various genotypes, and individual differences within pig populations. Additionally, some pigs experience serious side effects after vaccination, including fever, abortion, skin lesions, swollen joints, and persistent infections, etc [44,45,46,47]. In recent times, the commercial vaccines ASFV-G-ΔI177L and ASFV-G-ΔMGF, developed in Vietnam, which were reported to have few adverse effects and have a high protection rate, were found to be ineffective against a newly emerging highly virulent ASFV [48]. Although cell subculturing attenuates the infectivity and virulence of ASFV, its decreased antigenicity led to ineffective protection during pig immunization [47,49,50]. With the revelation of the ASFV gene’s mechanism of action, gene knockout became a crucial strategy in designing attenuated vaccines. Various knockout strains for vaccine development trials included Benin 97/1/MGF [51], OURT88/3I329L [52], and ASFV-GZΔI177LΔCD2vΔMGF [53], etc. However, since gene knockouts of ASFV may lead to antigenic changes, attenuated vaccines that have been developed exhibit inadequate cross-immunoprotection. Additionally, widespread attenuated vaccine immunization could pose a danger of heightened virulence [47]. Consequently, attenuated vaccines have so far not been used on a large scale worldwide due to safety concerns.

Overall, current ASFV vaccines fail to achieve both effectiveness and safety (Table 1), essentially due to the complexity and strong immunosuppressive capacity of ASFV. Invasion by ASFV may involve multiple viral and cellular receptor proteins [54]; ASFV primarily infects monocytes/macrophages and can be transmitted via apoptotic bodies within macrophages, causing antibodies ineffective in neutralizing the virus [55]; also, the production of antibodies against ASFV may induce an antibody-dependent enhancement (ADE) that facilitates viral infection [56]. Inactivated vaccines and subunit vaccines primarily activate the humoral immune response, making it difficult to produce effective defenses against ASFV. Attenuated vaccines can provide enhanced protection because, in addition to activating humoral immunity, they also induce powerful cellular immunity, which is capable of clearing ASFV infections. Nonetheless, attenuated vaccines retain residual virulence and may induce immunosuppression in pigs; moreover, the ASFV is capable of gene mutation and viral recombination [57], and the application of attenuated vaccines poses the risk of regaining virulence and the emergence of novel strains, thereby complicating safety assurances. At present, several vector vaccines for ASFV are being developed. Although vector vaccines can enter cellular replication and activate a cellular immunological response, the clinical immunoprotection remains suboptimal [58]. The reason for this may be that the chosen ASFV antigen fails to produce adequate cellular immune protection. Furthermore, given the large number of ASFV proteins, selecting the appropriate antigenic proteins or their combinations is quite challenging. In summary, the key to the development of a safe and effective ASFV vaccine depends on the in-depth study of ASFV specific T-cell immunity mechanisms.

A critical synthesis of these vaccine development failures reveals that they are not merely setbacks in formulation but are fundamentally attributable to the sophisticated immune evasion mechanisms wielded by ASFV. The inadequacy of inactivated and subunit vaccines underscores the limitations of a humoral immunity-centric approach against a virus that adeptly avoids antibody neutralization via macrophage tropism, ADE, and rapid mutation. Similarly, the safety concerns and inconsistent efficacy of attenuated vaccines highlight the inherent risk of retaining viral genes that encode immunomodulators, which can suppress host responses even in an attenuated context. This unique challenge—the need to counteract a virus armed with an arsenal of proteins that actively dismantle both innate and adaptive immunity—distinguishes ASFV vaccine development from that of many other pathogens. Consequently, these insights powerfully guide the rationale for epitope-focused strategies. By moving beyond whole-virus or whole-protein approaches, next-generation vaccines can be rationally designed to circumvent these evasion tactics. This involves precisely targeting conserved and immunodominant T-cell epitopes to elicit robust, multispecific cytotoxic T-cell responses that are capable of recognizing and clearing infected cells, irrespective of the virus’s antibody evasion strategies, thereby offering a more promising path toward a safe and effective vaccine.

## 2. T-Cell Immunity in ASFV Infection

Traditional veterinary vaccines have mostly concentrated on inducing humoral immunity, but have insufficiently focused on the significance of cellular immunity [59]. However, it has been shown that most ASFV protective antigens are inadequate for ensuring comprehensive protection [60] and that neutralizing antibodies have limited efficacy against strong strains of ASFV [37,40,61]. More importantly, cell-mediated immune responses play an essential role in resistance to ASFV [61,62,63].

At the end of the 20th century, a study indicated that experimental pigs infected with avirulent or attenuated strains of ASFV activated specific T-cell proliferation in vivo [64]. Subsequently, several experiments in vitro revealed that swine peripheral blood mononuclear cells (PBMC) proliferate and produce interleukin-2 after infection with homologous or heterologous ASFV [63,65,66]. Martins et al. [67] showed the importance of SLA-I in cytotoxic T lymphocyte (CTL) activity during AFSV infection through monoclonal antibody blocking experiments. Oura et al. [62] demonstrated that the efficacy of the attenuated vaccine OURT88/3 in protecting pigs against homologous virus strains was closely associated with CD8+ T lymphocytes through CD8+ T cell depletion tests. Fan et al. [68] found a transient increase in the total number of T lymphocyte subsets, including helper T cells (Th), CTLs, and double-positive T cells (DP-T cell), after the immunization of pigs with the attenuated vaccine HLJ/18-7GD. These findings indicate that specific CTL responses are crucial in defending against ASFV infection. However, ASFV significantly impairs T-cell immunity. Tian et al. [28] discovered that T-cell subsets in pigs infected with ASFV CADC_HN09 were fully activated and expressed IFN-γ 5 days post-infection; nevertheless, subsequent immunosuppression diminished the capacity of T cells to produce cytokines. Hühr et al. [69] reported that T-cell immune responses were impaired, and the total number of CD4+ T cells, DP-T cells, and CD8+ T cells decreased in domestic pigs infected with the highly virulent ASFV Armenia08.

The T-cell epitope is a short peptide presented by MHC-I or a longer peptide presented by MHC-II and can be recognized by the T Cell Receptor (TCR). Attenuated vaccines possess adequate T-cell epitopes, making it possible to provide effective protection, but they also include quantities of virulence proteins that may result in immunosuppression. If sufficient T-cell epitopes in ASFV are identified and a vaccine is developed accordingly, it may successfully activate the T cell immune response while preventing immunosuppression, thus enabling an optimal vaccine that is both safe and effective. Yet identification of ASFV T-cell epitopes remains very limited.

## 3. Identification of ASFV T-Cell Epitopes

**Figure 1:**
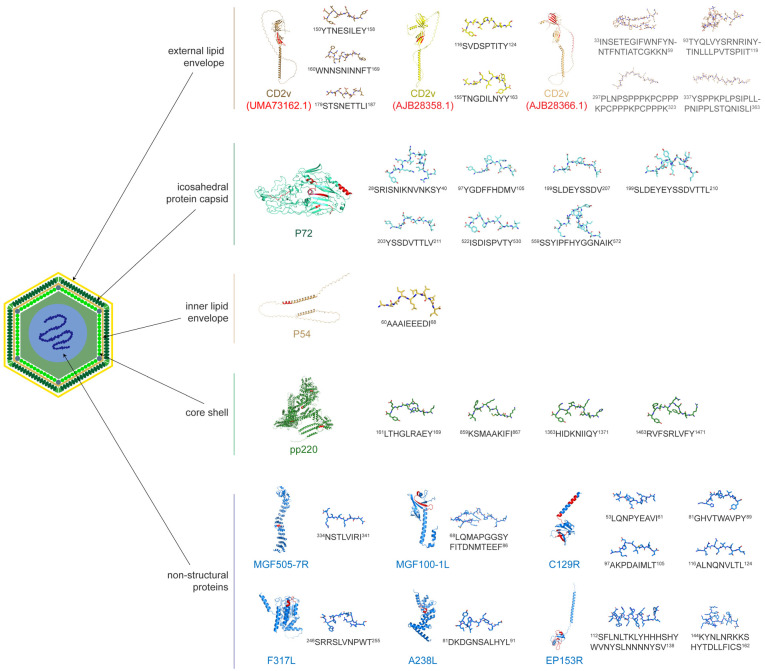
T-cell epitopes derived from different proteins of ASFV. The identified epitopes of ASFV are shown in the virus particle. The structures of the relevant proteins and peptides are predicted by AlphaFold3 (alphafoldserver.com, access on 11 July 2025) and visualized using PyMOL (Version: 3.1.1). The positions of epitopes in the protein are indicated in red, and the amino acid compositions of the epitopes are represented in stick mode. The CD2v protein is highly variable, and the identified epitopes are derived from three different CD2v proteins (accession numbers from the NCBI GenBank database).

The traditional T-cell epitopes of ASFV identified to date are listed in Table 2, including structural proteins located in various layers of the ASFV as well as some non-structural proteins; all of them have been demonstrated to induce T-cell immune responses. CD2v in the external lipid envelope exhibits high variability and has the largest number of identified epitopes. Among the three variants, a total of nine epitope peptides have been identified, five of which have lengths ranging from eight to eleven amino acids, while the other four epitopes, derived from the virus strain AJB28366.1, are excessively long and may contain multiple T-cell epitopes. p72, in the icosahedral protein capsid, has had up to seven epitope peptides identified, among which ^199^SLDEYSSDVTTL^210^ shares partial sequence overlap with both ^199^SLDEYSSDV^207^ and ^203^YSSDVTTLV^211^. Only one epitope peptide was identified in the p54 protein, located in the inner lipid envelope, and four epitope peptides were identified in the pp220 protein, situated in the core shell. In addition, a total of ten epitopes were identified in the non-structural proteins F317L, C129R, MGF505-7R, MGF100-1L, A238L, and EP153R. Among them, both MGF505-7R and EP153R have been identified to have long epitopes, which may contain one or more potential SLA-I-restricted T-cell epitopes (Figure 1).

Huang et al. [70] confirmed that F317L can effectively activate cellular immunity through IFN-γ Enzyme-Linked Immunospot (IFN-γ ELISpot) assays and flow cytometry; they predicted and synthesized 29 T-cell epitopes to stimulate mouse splenic lymphocytes, and subsequent IFN-γ ELISpot experiments demonstrated that F25 (^246^SRRSLVNPWT^255^) induced a stronger T-cell immune response than the F317L protein. Zhai et al. [71] showed that C129R activated both cellular immunity and humoral immunity, identifying four T-cell epitope peptides of C129R; among these, C14 (^81^GHVTWAVPY^89^) exhibited greater conservation across ASFV genotype I and II strains, and C11 (^53^LQNPYEAVI^61^) significantly enhanced T-cell immune responses. Zajac et al. [72] identified 4 CD8+ T-cell epitope peptides, p34 (^161^LTHGLRAEY^169^), p37 (^859^KSMAAKIFI^867^), p150 (^1363^HIDKNIIQY^1371^), and p150 (^1463^RVFSRLVFY^1471^), establishing that these epitopes were 100% conserved in various ASFV genotypic strains isolated from domestic pigs, wild boars, warthogs, and ticks. Song et al. [73] utilized NetMHCpan-4.0 and mouse splenic lymphocyte IFN-γ ELISpot to validate two T-cell epitopes on p72 (^522^ISDISPVTY^530^) and CD2v (^150^YTNESILEY^158^); Song et al. [74] identified a T-cell epitope on CD2v (^160^WNNSNINNFT^169^) and established that this peptide was sequence-identical and highly conserved across eight distinct ASFV strains. Argilaguet et al. [75] confirmed two T-cell epitopes from CD2v (^116^SVDSPTITY^124^, ^155^TNGDILNYY^163^) based on their binding affinity to TAP, utilizing a support vector machine regression model for prediction and IFN-γ ELISpot assays with pig fibroblasts and PBMCs. Camós et al. [76] obtained 106 peptide sequences using MHC immunoaffinity chromatography (MHC-IAC), subsequently identifying two more precise T-cell epitopes from MGF505-7R (^334^NSTLVIRI^341^) and A238L (^81^DKDGNSALHYL^91^) via IFNγ-ELISpot; the team also recognized a 19-mer peptide in MGF100-1L (^68^LQMAPGGSYFITDNMTEEF^86^) through online prediction and IFNγ-ELISpot. Yue et al. [77] revealed that peptides from CD2v (^179^STSNETTLI^187^), p54 (^60^AAAIEEEDI^68^), and p72 (^97^YGDFFHDMV^105^, ^199^SLDEYSSDV^207^, ^203^YSSDVTTLV^211^) could co-complex with SLA-1 *0101 in vitro, refold to form stable MHC–peptide (pMHC) complexes, and identified SLDEYSSDV and YSSDVTTLV as immunodominant epitopes using a flow cytometry assay based on SLA tetramer. Sun et al. [78] discovered three T-cell epitopes on p72 (^199^SLDEYSSDVTTL^210^, ^28^SRISNIKNVNKSY^40^, ^559^SSYIPFHYGGNAIK^572^) using in vitro synthesis of SLA–peptide (pSLA) tetramers and flow cytometry analysis. Burmakina et al. [79] found six discrete T-cell epitope regions on CD2v and EP153R (I-VI) in the PBMCs of pigs inoculated with the ASFV KK-262 strain, using the IFN-γ ELISpot assay.

**Table 2:** Identification of ASFV T-cell epitopes.

Proteins	Amino Acid Sequence	SLA	Predicting Tools	Experimental Animal	Experimental Methods	Cite
F317L	^246^SRRSLVNPWT^255^	/	IEDB; NetMHCpan-4.1	Mouse	IFN-γ ELISpot	[69]
C129R	^53^LQNPYEAVI^61^; ^81^GHVTWAVPY^89^; ^97^AKPDAIMLT^105^; ^116^ALNQNVLTL^124^	SLA-1*0401 SLA-2*0401 SLA-3*0401	IEDB; NetMHCpan-4.1	Mouse	IFN-γ ELISpot	[70]
pp220	^161^LTHGLRAEY^169^; ^859^KSMAAKIFI^867^; ^1363^HIDKNIIQY^1371^; ^1463^RVFSRLVFY^1471^	/	NetMHCpan-2.8	Pig	IFN-γ ELISpot	[71]
CD2v	^150^YTNESILEY^158^	SLA-1*0401	NetMHCpan-4.0	Mouse	IFN-γ ELISpot	[72]
CD2v	^160^WNNSNINNFT^169^	/	ABCpred	Mouse	IFN-γ ELISpot	[73]
CD2v	^179^STSNETTLI^187^	SLA-1*0101	NetMHCpan-4.0	Pig	Refold in vitro	[76]
CD2v	^116^SVDSPTITY^124^; ^155^TNGDILNYY^163^	/	TAPREG	Pig	IFN-γ ELISpot	[74]
CD2v	^33^INSETEGIFWNFYNNTFNTIATCGKKN^59^; ^93^TYQLVYSRNRINYTINLLLPVTSPIIT^119^; ^297^PLNPSPPPKPCPPPKPCPPPKPCPPPK^323^; ^337^YSPPKPLPSIPLLPNIPPLSTQNISLI^363^	/	IEDB	Pig	IFN-γ ELISpot	[78]
MGF100-1L	^68^LQMAPGGSYFITDNMTEEF^86^	/	NetMHCpan-3.0	Pig	IFN-γ ELISpot	[75]
MGF505-7R	^334^NSTLVIRI^341^	/	/	Pig	MHC-IAC; IFN-γ ELISpot	[75]
A238L	^81^DKDGNSALHYL^91^	/	/	Pig	MHC-IAC; IFN-γ ELISpot	[75]
p54	^60^AAAIEEEDI^68^	SLA-1*0101	NetMHCpan-4.0	Pig	Refold in vitro	[76]
p72	^522^ISDISPVTY^530^	SLA-1*0401	NetMHCpan-4.0	Mouse	IFN-γ ELISpot	[72]
p72	^97^YGDFFHDMV^105^; ^199^SLDEYSSDV^207^; ^203^YSSDVTTLV^211^	SLA-1*0101	NetMHCpan-4.0	Pig	Tetramers	[76]
p72	^199^SLDEYSSDVTTL^210^	SLA-1*0101	IEDB	Pig	Tetramers	[77]
p72	^28^SRISNIKNVNKSY^40^	SLA-3*0301				
p72	^559^SSYIPFHYGGNAIK^572^	SLA-1*1201				
EP153R	^112^SFLNLTKLYHHHSHYWVNYSLNNNNYSV^138^; ^144^KYNLNRKKSHYTDLLFICS^162^	/	IEDB	Pig	IFN-γ ELISpot	[78]
pp220	IADAINQEF; FLNKSTQAY; QIYKTLLEY; SLYPTQFDY	SLA-1*0401	NetMHCcon; IEDB; ToxinPred; GalaxyPepDock; GalaxyRefine- Complex; PRODIGY; MDWeb	/	/	[79]
pp62	GTDLYQSAM; FINSTDFLY; STDFLYTAI	SLA-1*0401		/	/	
G1211R	AADDTTCYY	/	IEDB: NetMHCpanEL 4.1	Mouse: Pig	/	[80]
	MPIDIHEVRY					
CP2475L	HIDKNIIQY					
CP204L	VVFHAGSLY					
CP530R	YSDPETVHSY					
NP1450L	ILDLIRLQY					
D339L	SVYHVQEEL					
D1133L	VPAKPEHLY					
D345L	HIDGTYLGY					
P1192R	MPVYQELGY					
H359L	IPDISFVGY					
E423R	SEYKQYNEF					
Q706L	IVDEAHNLF					
E248R	FIADAISAV					
I329L	ISFSNNNTY					

However, the identification of these T-cell epitopes presents some limitations. The experiments of Huang, Song, and Zhai et al. used ASFV proteins to immunize mice; hence, the subsequent experiments demonstrated activation and potentiation of the epitope peptide on the T-cell immune response, assessed only in mice and not in pigs. Herrera et al. [80] discovered six potential CD8+ epitope peptides by prediction, structural modeling, and computational data analysis, yet these findings lacked empirical testing. Sun et al. [81] identified several ASFV T-cell epitopes and developed a multi-epitope nanoparticle vaccine utilizing these epitopes that could induce T-cell immunity. Additionally, there was an overlap between the B-cell and T-cell epitopes among the identified epitopes; however, the efficacy of each specific epitope in eliciting T-cell immunity remains ambiguous.

A critical synthesis of the identified epitopes reveals several promising candidates to prioritize for future vaccine development. Epitopes derived from highly conserved and essential structural proteins, such as the major capsid protein p72 and the polyproteins pp220, are particularly attractive due to their low propensity for mutation and critical role in virion integrity. For instance, the p72 epitopes SLDEYSSDV and YSSDVTTLV have been consistently identified across independent studies using robust methodologies (tetramer staining, INF-γELISpot) and demonstrate immunodominance. Similarly, epitopes from pp220 show 100% conservation across diverse ASFV genotypes and strong immunogenicity. Among non-structural proteins, epitopes from C129R also emerge as strong candidates due to their role in immunomodulation and demonstrated ability to stimulate potent T-cell responses. While numerous epitopes have been identified in variable proteins like CD2v, their importance may be limited by strain-specificity.

The identification of ASFV T-cell epitopes has advanced, yet the total quantity remains scarce and is concentrated on just a few ASFV proteins, which is perhaps insufficient for vaccine development requirements. The identification of ASFV T-cell epitopes demands surmounting numerous theoretical and technical obstacles.

## 4. Challenges in ASFV T-Cell Epitope Identification

### 4.1. SLA Polymorphism

T-cell epitopes are MHC-restricted and depend on SLA for effective presentation in swine. SLA exhibits high polymorphism, and allele expression differs among individuals [82]. The immunopolymorphism database IPD-MHC presently comprises 262 SLA-I sequences and 216 SLA-II sequences (https://www.ebi.ac.uk/ipd/mhc/) (accessed on 13 April 2025). The NCBI GenBank contains approximately 1000 SLA-I, whereas the α and β chains of SLA-II collectively amount to around 400 sequences (https://www.ncbi.nlm.nih.gov/genbank/) (accessed on 13 April 2025).

**Figure 2:**
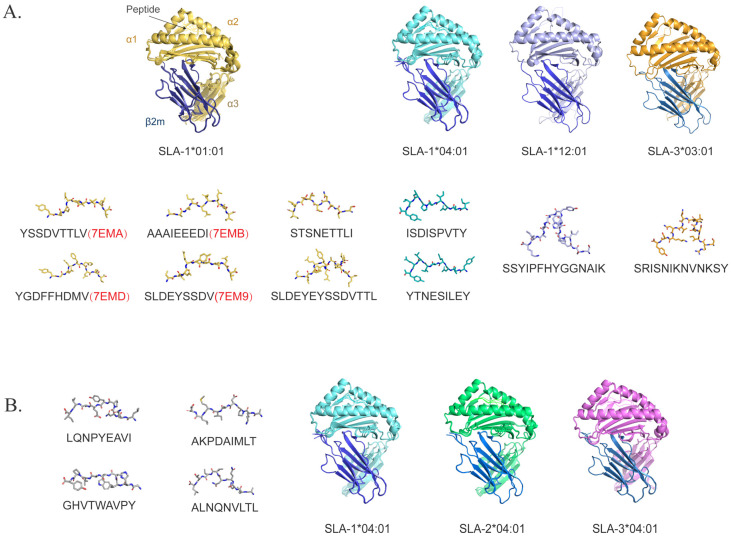
ASFV epitopes presented by different SLA-I. The heavy chain of different SLA-I molecules is shown in various colors, consisting of α1, α2, and α3 domains. The peptide is located in the PBG between the α1 and α2 domains. The light chain β2m is in dark blue. The complex structures of four SLA-1*0101 with ASFV epitopes were retrieved from the PDB database, whereas the remaining structures of SLA-I molecules and ASFV epitopes were predicted using AlphaFold3 (alphafoldserver.com, access on 15 July 2025). (**A**) The ASFV epitope peptides are presented by defined SLA-I. The complex structures of each of the SLA-I and ASFV epitope peptides are superimposed above, whereas the distinct bound epitopes are displayed individually below. The PDB accession codes of the four pSLA complexes with resolved structures are highlighted in red. (**B**) The four ASFV peptides on the left may be the restricted epitopes of SLA-1*04:01, SLA-2*04:01, and SLA-3*04:01, but their corresponding relationships with these three SLA-I molecules have not been clarified.

The SLA-I consists of heavy and light chains, with its highly variable regions predominantly situated in the heavy chain α1 and α2 structural domains, particularly within the peptide binding groove (PBG) that binds directly to the peptide [83]. The primary distinction of SLA-II from SLA-I is its presentation as a heterodimer of an α chain and β chain. Analogous to HLA (human leukocyte antigen) class II, SLA-II demonstrates high polymorphisms at SLA-DRB1 and SLA-DQB1, with comparatively few mutations in SLA-DRA [84]. Polymorphic residues inside the PBG result in variations in the sequence, length, and conformation of peptides bound by diverse SLA [84,85]. Consequently, different SLA sequences among individuals exhibit different peptide binding specificities, and highly variable sites may bind various antigenic peptides. The extensive polymorphism of SLA in pigs results from natural selection, enhancing the diversity of bindable epitopes, thus improving infectious resistance. However, it complicates the identification of pathogen epitopes. The polymorphism of SLA necessitates that vaccine development encompasses a diverse array of epitopes from various SLA haplotypes to ensure the vaccine’s extensive protective efficacy. Currently, the identified ASFV epitopes that map to distinct SLA-I molecules include six epitopes for SLA-1*01:01, two epitopes for SLA-1*04:01, as well as one epitope each for SLA-1*12:01 and SLA-3*03:01. Among these, the structures of four epitope peptides in complexes with SLA-1*01:01 have been resolved (PDB ID: 7EMB, 7EMA, 7EMD, 7EM9) (Figure 2A). Additionally, four epitope peptides have only been predicted to bind to SLA-1*04:01, SLA-2*04:01, and SLA-3*0401, while their actual binding capacity to each SLA-I molecule remains unelucidated (Figure 2B).

On the other hand, effective T-cell epitopes are dispersed among the numerous proteins of ASFV. Direct screening using techniques like synthetic peptide libraries would be prohibitively expensive in terms of both time and cost, in addition to being susceptible to individual variations in experimental pigs. Thus, the successful screening of T-cell epitopes of ASFV requires the accumulation of substantial data on SLA and the elucidation of the conformational patterns of PBG. Only a limited number of parsing SLA-I crystal structures and peptide presentation characteristics have been so far reported [86,87,88], while the crystal structure of SLA-II remains unreported. More studies on crystal structure and peptide presentation characteristics of SLA are required for further precise screening and identification of T-cell epitopes.

In summary, overcoming the hurdle of SLA polymorphism will require a concerted effort to decipher the molecular details of peptide presentation. A priority is to solve high-resolution crystal structures of SLA-I and, crucially, SLA-II molecules from various common haplotypes in complexes with diverse peptides. This will elucidate the precise physicochemical features of the peptide-binding groove that dictate binding specificity. The insights gained, such as key anchor residues and binding motifs, will be instrumental in developing predictive models with greatly enhanced accuracy. Furthermore, this structural knowledge will enable the rational design of promiscuous epitopes or epitope strings that can bind to multiple SLA alleles, ultimately guiding the development of broadly protective T-cell vaccines against ASFV.

### 4.2. The Limited Prediction Tools

To date, numerous advanced epitope prediction tools are available, including NetMHCpan [89], POPISK [90], PAAOD [91], TEPITOPE [92], SYFPEITHI [93], MHCflurry [94], MixMHCpred [95], etc. The primary online tools for predicting T-cell epitopes in swine are IEDB, NetMHCpan, and MixMHCpred (Table 3).

The IEDB is currently the most widely used online tool for T-cell epitope prediction, providing experimental data from a variety of fields and allowing large-scale data to be analyzed using a diverse range of methods [96]. It also presents three-dimensional structural data for pMHC, supplying computational insights into intermolecular interactions [97,98]. The 2024 version of the Next-Generation IEDB Tools consolidates all epitope-related functionalities from the previous IEDB, including MHC binding, elution, immunogenicity, and processing, thus enhancing accessibility and usability [98].

NetMHCpan’s initial training data comprised solely peptide–HLA complexes [99]. NetMHCpan-2.0 covered MHC-I data from various species, including swine, demonstrating high accuracy in predicting the binding sequence of SLA-1*0401 [100]. NetMHCIIpan-3.0 utilizes pseudo sequences to enhance the precision of peptide binding environment predictions [101], while NetMHCpan-3.0 enhances ligand identification accuracy through a more advanced full-allele/full-length algorithm [102]. Updated so far, NetMHCIIpan-4.0 and NetMHCpan-4.1 amalgamate diverse training data types to predict MHC ligands identified by mass spectrometry (MS), yielding more precise results [103].

MixMHCpred attains superior prediction accuracy utilizing extensive MS eluted peptide data [104]. Its predictive performance for HLA-I binding to peptides across various alleles is consistently enhanced with successive versions [105]. Particularly precise predictions are possible for diverse alleles exhibiting analogous sequences at the binding site [106]. MixMHCpre is now capable of executing cross-species predictions, encompassing SLA-restricted epitopes.

Nevertheless, current prediction tools and methods are based on algorithmic construction and scoring of HLA and lack data relevant to swine SLA, thus predicting SLA-restricted epitopes with low accuracy [105,107,108].

Alphafold was developed by DeepMind in 2021 based on a neural network model, with the subsequently enhanced Alphafold3 in 2024 [109]. It has significantly advanced the precision of protein complex structure prediction and are expected to serve as a new instrument for the structural prediction of TCR-pMHC complexes [110,111]. Several recent structural predictions based on Alphafold have demonstrated potential for peptide prediction of animal MHC epitopes. Wang et al. [112] utilized it to predict the complex structure of pMHC binding across various alleles in bats; Tang et al. [113] predicted the peptide-binding platforms formed by duck MHC α1 and α2 structural domains using Alphafold2, subsequently validating hydrogen bonding between the peptide and PBG through experimental methods. AlphaFold3′s precision in predicting protein structure interactions offers a prospective resolution to the challenges of epitope prediction for MHC across various species and alleles.

The accuracy gap in epitope prediction for swine can be bridged by leveraging and contributing to iterative improvements in bioinformatic tools. Firstly, the integration of structural prediction tools like AlphaFold3, which can model pSLA complex structures with high confidence, offers a revolutionary approach to in silico screening by assessing potential peptide–MHC interactions in a structural context. Secondly, it is imperative to establish a community standard where newly identified ASFV T-cell epitopes, along with their corresponding SLA restriction and experimental validation data, are consistently deposited into public repositories like IEDB. This expanding dataset will provide the essential training material needed to refine the algorithms of NetMHCpan, MixMHCpred, and other platforms, specifically for SLA molecules, creating a positive feedback loop for enhanced prediction performance.

To facilitate standardized and efficient epitope prediction for ASFV, we propose a practical workflow: First, determine the predominant SLA class I and II alleles in the target swine population through genotyping. Second, select antigenic proteins from ASFV based on conservation and immunogenicity evidence, and retrieve their full-length amino acid sequences from reliable databases (e.g., NCBI Protein). Third, input the protein sequences into prediction tools (e.g., NetMHCpan for SLA-I, NetMHCIIpan for SLA-II) for the identified SLA alleles. Fourth, filter and rank the predicted peptides based on their binding affinity scores (typically %Rank or IC50 value), prioritizing those with high predicted binding. Finally, integrate other filters such as peptide conservation across ASFV strains and exclusion of potential cross-reactivity with the swine proteome to generate a final list of high-priority candidate epitopes for subsequent experimental validation.

### 4.3. Experimental Methods

Prediction tools can conduct initial screening of T-cell epitope peptides of ASFV, but their validity as true ASFV epitopes requires experimental verification. At present, there are three main experimental validation methods for ASFV epitope identification, including in vitro stimulation of immune cells with epitope peptides to detect the production of IFN-γ; in vitro refolding of major histocompatibility pMHC complexes and construction of tetramers to monitor specific T-cell responses and the immunoreactivity of peptides; and MHC-IAC, using specific monoclonal antibodies to capture pSLA complexes from infected tissues, followed by peptide ligand elution and liquid chromatography-tandem mass spectrometry (LC-MS/MS) analysis.

**Figure 3:**
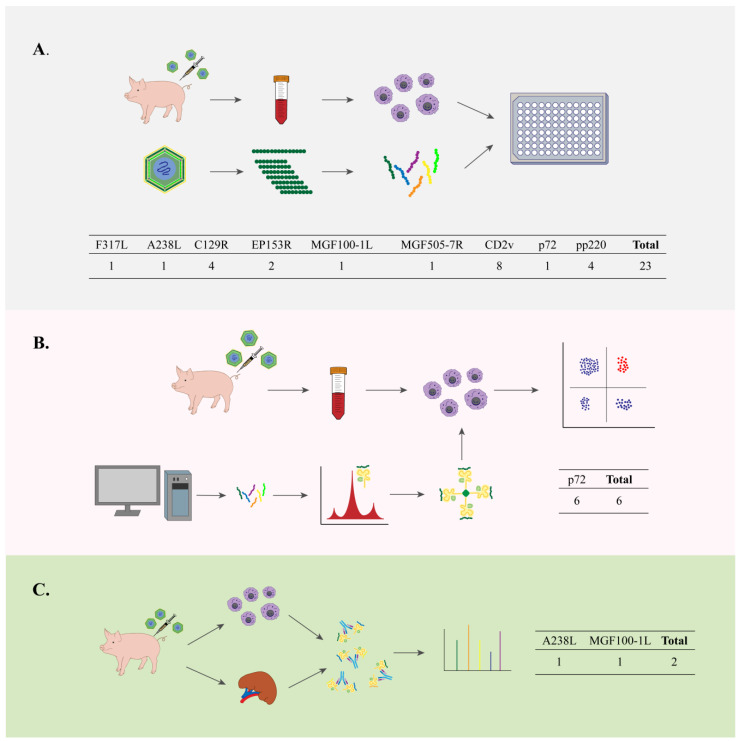
Three experimental methods to identify ASFV T-cell epitopes. (**A**) IFN-γ ELISpot. A peptide library is first synthesized; then PBMCs are obtained from infected swine; and finally, the epitopes are identified by stimulating PBMCs with the peptide and detecting IFN-γ. Currently, 23 epitopes have been identified by this method, but some of the PBMCs used for epitope identification were from mice. (**B**) Tetramers. Possible epitopes are first predicted online, then refolded with SLA and constructed as tetramers in vitro; PBMCs are obtained from infected swine; finally, using tetramers to stain PBMCs, epitopes are identified by flow cytometry. Six epitopes from the p72 protein were identified by this method. (**C**) MHC-IAC. pSLA complexes are captured in infected swine tissues and cells using SLA monoclonal antibodies; SLA-restricted peptides are finally eluted and analyzed by LC-MS/MS. Only two epitopes of ASFV have been validated by this method.

ELISpot is exceptionally sensitive and can detect the frequency of immunomodulatory mediators released by specific immune cells following stimulation. Since T cells release cytokines such as IFN-γ upon binding to T-cell receptors and their antigenic epitopes, the IFN-γ ELISpot may quantify antigen-specific T-cell production [114,115]. Therefore, this method has been employed to identify ASFV epitopes [70,71,72,73,74,75,76,79,81]. Among the three methods, IFN-γ ELISpot identified the most epitopes from ASFV, including nine proteins with 23 epitopes. Among them, two epitopes, respectively, located in MGF505-7R and A238L, were validated by MHC-IAC and subsequently re-validated by IFN-γ ELISpot (Figure 3A).

Constructing pMHC tetramers and flow cytometry represent the gold standard for identifying specific T-cell epitopes. The procedure involves utilizing MHC with peptides for protein expression and in vitro folding, constructing tetramers and staining PBMCs from infected animals, and subsequently collecting the PBMCs and analyzing them by flow cytometry. Tetramer construction technology has been applied to identify T-cell epitopes of ASFV [77,78]. As of now, only six epitopes derived from the p72 protein have been validated by tetramer staining and flow cytometry (Figure 3B). Nonetheless, as previously noted, there are fewer studies on SLA-II, and the stability of SLA-II in vitro is poor. Therefore, no use of SLA-II tetramers has been reported.

MHC-IAC relies on the analysis of MHC structure and the development of specific antibodies, having been extensively utilized in human medicine for screening T-cell epitopes, but to a lesser extent in swine diseases. The pMHC complex is captured through immunoaffinity chromatography of diseased tissues or cells from infected animals using a monoclonal antibody specific to SLA. It is subsequently eluted with acetic acid, purified, and analyzed via LC-MS/MS to separate the peptides in a linear gradient, followed by data processing for screening of T-cell epitope [116,117]. Only two ASFV epitopes have been identified by MHC-IAC, at A238L and MGF100-1L (Figure 3C). There is a scarcity of studies on SLA-II and a deficiency of broad-spectrum, high-affinity antibodies, resulting in the absence of this method for identifying swine CD4+ T-cell epitopes.

To address the inconsistencies in epitope validation, the field would benefit greatly from establishing a standardized, multi-tiered experimental pipeline. This pipeline should begin with in silico prediction and MHC binding affinity assays, proceed to in vitro functional validation using primary pig cells, and culminate in in vivo validation in adoptive transfer or challenge studies in pigs for the most promising candidates. Widespread adoption of such a standardized framework would ensure the reliability and comparability of newly identified epitopes, break the current situation, and establish a rigorous benchmark for claiming a bona fide ASFV T-cell epitope. This is a critical step toward building a robust foundation for vaccine development.

## 5. ASFV T-Cell Epitope Vaccine

### 5.1. Lessons from Other Vaccine Platforms

Experience gained from the development of novel vaccines against other porcine viruses provides valuable insights for ASFV vaccine development, particularly regarding multi-epitope tandem vaccines and in-depth studies on T-cell immune mechanisms. For instance, the challenges encountered during the development of effective vaccines against porcine reproductive and respiratory syndrome (PRRS) underscore the critical importance of inducing robust cellular immunity, while also revealing the limitations of inactivated and live-attenuated vaccines—such as their general inability to provide cross-protection and their potential safety risks. Currently, an effective strategy for enhancing the immunogenicity of synthetic peptide vaccines against PRRSV involves the tandem arrangement of multiple epitopes combined with suitable adjuvants. Furthermore, a deeper understanding of the spatial conformation of PRRSV and the characteristics of the host immune system in pigs is essential for advancing the development of such synthetic peptide vaccines [118]. Similarly, extensive research on foot-and-mouth disease (FMD) peptide vaccines has confirmed that only by incorporating multiple B-cell and T-cell epitopes can broad-spectrum protection be achieved and antigenic variability be addressed [119]. Current vaccines against swine influenza A virus (IAV-S) struggle to cope with continuous viral variation, fail to provide broad protection, and must be tailored to specific strains circulating in target populations. There is a relative lack of research on vaccine-induced antibody and T-cell immune responses in pigs. Further exploration in this area will help identify immunodominant B-cell and T-cell epitopes [120].

### 5.2. mRNA Vaccines

mRNA vaccines have demonstrated significant success in pandemic prevention in recent years [121]. mRNA vaccines exhibit great safety and efficacy, can be scaled up for manufacture [122], and induce strong cellular protection through the intracellular translation of antigenic proteins [123]. Currently, several mRNA vaccines targeting infectious diseases are being developed, for example, the 20-HA mRNA-LNP, encoding 20 hemagglutinin antigens from influenza A and B [124]; the Lyme disease mRNA vaccine, encoding 19 salivary proteins from I. scapularis and producing an effective immune response in guinea pigs [125]; and the PEDV-S mRNA-LNP vaccine and the PDCoV-S mRNA-LNP vaccine, both encoding the complete spike protein and inducing both humoral and cellular immunity in piglets [126,127]. Due to the Coronavirus Disease 2019 (COVID-19) pandemic, two mRNA vaccines, Comirnaty and Spikevax, received emergency use authorization within a year and were subsequently deployed following clinical trials [128].

Currently, research on mRNA vaccines targeting ASFV remains limited. Gong et al. [129] developed mRNA/Man-LNP, encoding the complete p30 protein of ASFV, which produces high IgG titers and activates CD4+ and CD8+ T cells in mice. Sira et al. [130] predicted multiple epitopes that induce cellular and humoral immunity based on 100 ASFV proteins, constructed a multi-epitope ASFV mRNA vaccine, and evaluated it through computational simulations. But it has yet to be tested in animals. Although there are fewer studies on mRNA vaccines for ASFV, their demonstrated efficacy in preventing various infectious diseases, particularly their powerful capacity to induce T cell immunity, indicates that mRNA vaccines may emerge as a primary focus for ASFV vaccine development.

### 5.3. Delivery Systems

Advanced delivery systems are crucial for the successful development of epitope-based vaccines. Epitope peptides themselves exhibit low immunogenicity and are susceptible to degradation by various enzymes in vivo. However, the combined use of vaccine delivery carriers and adjuvants can effectively enhance the immunogenicity of epitope peptides and protect them from degradation [131,132]. In recent years, nanoparticle carriers have attracted widespread attention in the development of novel vaccines and cancer immunotherapy due to their dual functionality in protecting antigenic peptides and exhibiting adjuvant activity.

Carriers such as poly (lactic-co-glycolic acid) (PLGA) nanoparticles can encapsulate epitope peptides, preventing their degradation by endogenous enzymes before internalization by antigen-presenting cells (APCs). Additionally, the particulate nature of these carriers enhances uptake by APCs, thereby strengthening T-cell immune responses and the production of neutralizing antibodies. PLGA has already been approved by the U.S. Food and Drug Administration (FDA) and the European Medicines Agency, providing a solid foundation for its application [133,134,135,136]. Furthermore, self-assembling protein nanoparticles based on ferritin can display multiple epitope peptides on their surface in a highly ordered manner, significantly enhancing the immunogenicity of antigenic peptides with very low heterogeneity [136,137].

For mRNA vaccines, lipid nanoparticle (LNP) delivery systems are indispensable. LNPs not only protect mRNA molecules and deliver them to the cytoplasm but also act as intrinsic adjuvants by activating innate immune pathways [138]. This process strongly activates APCs and creates an inflammatory microenvironment conducive to robust T-cell immune responses. With the maturation of nanoparticle carrier technology, the design and development of ASFV epitope-based vaccines have become feasible.

### 5.4. Current Status and Prospects of ASFV Epitope-Based Vaccines

Today, several ASFV epitope peptide vaccines have been tested in lab. Sun et al. [81] developed a multi-epitope nanoparticle vaccine, TEP-Spy-NPs, comprising 14 ASFV T-cell epitopes. They demonstrated high immunogenicity, inducing large quantities of IFN-γ and IL-2 from mouse splenocytes and porcine PBMCs. Song et al. [73] created a multi-epitope nanovaccine targeting dendritic cells (DC), NanoFVax, that includes all predicted T-cell and B-cell epitopes from p72, CD2v, pB602L, and p30, and has demonstrated the capacity to induce substantial T-cell responses alongside elevated antibody responses persisting for over 231 days. However, it should be noted that neither vaccine has undergone ASFV challenge protection experiments, so their actual protective efficacy remains unclear.

Based on the current research landscape, the next generation of ASFV T-cell vaccines should adopt integrated strategies focusing on three critical pathways: rational design of multi-epitope constructs to broaden immune recognition; utilization of advanced delivery platforms to enhance antigen presentation and stability; and incorporation of immunologic adjuvants to counteract viral immunosuppression. Among existing technologies, the mRNA-LNP platform is considered to be one of the most promising approaches. It effectively integrates optimized antigen design with inherent adjuvant properties, thereby robustly inducing potent T-cell immune responses.

It is particularly important to note that the ASFV genome encodes more than 150 proteins, containing a large number of potential antigenic epitopes. mRNA vaccines encoding only one or a few proteins are unlikely to induce comprehensive immune responses. Therefore, screening of immunodominant epitopes, design of multi-epitope mRNA, and integration with LNP delivery systems is expected to be a key direction for resolving the contradictions between breadth, potency, and safety in ASFV vaccines.

## 6. Conclusions

ASFV presents substantial hurdles to vaccine development, largely attributed to its robust immunosuppressive activity and the extensive repertoire of proteins it encodes. Among existing research strategies, vaccine development based on ASFV T-cell epitope identification emerges as a particularly advantageous approach: it enables precise targeting of epitopes associated with protective immunity while circumventing safety concerns—such as virulence reversion—that are intrinsic to attenuated vaccines. Nevertheless, critical gaps persist in this field: the quantity of identified T-cell epitopes remains inadequate, with considerable variability in their accuracy, preventing them from meeting the demands of effective vaccine design.

The identification of ASFV T-cell epitopes is primarily constrained by three core bottlenecks. First, current identification methodologies struggle to fully account for the polymorphism of SLA—a critical factor governing epitope presentation. Second, epitopes identified through ex vivo stimulation, though capable of activating mouse splenocytes or PBMCs, fail to accurately mirror the actual epitopes displayed on the surface of infected cells in vivo; this limitation, in turn, hinders the prediction of optimal immunogenic responses. Third, while in vitro tetramer construction and flow cytometry are reliable tools for epitope identification, they impose high demands on experimental techniques and hardware, thereby restricting their widespread use. Furthermore, although MHC-IAC serves as a relatively mature platform for SLA epitope identification, its practical application remains significantly constrained by the scarcity of broad-spectrum, high-affinity antibodies.

In the practical development of epitope-based vaccines, vector vaccines and mRNA vaccines have demonstrated clear potential. Nanoparticle-carrier epitope vaccines and multi-epitope mRNA vaccines have both entered the preliminary exploration phase, with mRNA vaccines standing out for their distinct advantages. As validated in the control of COVID-19, mRNA vaccines can induce robust T-cell immunity via intracellular antigen translation and exhibit high safety profiles—traits that align closely with the core requirements for ASFV vaccines (i.e., strong cellular immunity and safety)—positioning mRNA vaccines as a highly feasible new direction in ASFV vaccine research.

To address the aforementioned limitations and advance the development of ASFV T-cell epitope vaccines, future studies should focus on three prioritized, actionable areas. First, it is essential to elucidate the molecular mechanisms underlying ASFV-specific T-cell immunity: this involves clarifying the functional roles of different T-cell subsets in protective immunity, as well as the characteristics of immunodominant epitopes, which will provide a theoretical foundation for screening core protective epitopes. Second, efforts should be directed toward overcoming the bottleneck of SLA polymorphism coverage and optimizing epitope identification protocols. Specific measures include developing broad-spectrum, high-affinity antibodies to enhance the applicability of MHC-IAC; integrating multi-omics data to refine in silico epitope prediction algorithms, thereby improving the accuracy of candidate epitope screening; and establishing standardized validation workflows to ensure the reliability of identified epitopes. Third, the integration of multi-epitope design with advanced delivery technologies should be promoted. Given the complexity of ASFV antigens, single-epitope vaccines are unlikely to elicit comprehensive protection; thus, rational design of multi-epitope constructs (incorporating immunodominant T-cell and B-cell epitopes) is necessary. Concurrently, optimizing delivery systems—such as enhancing the efficiency of LNPs in delivering mRNA-encoded multi-epitopes and boosting the adjuvant activity of PLGA nanoparticles—will help balance the immunogenicity and safety of vaccines.

In summary, the formidable challenge of developing an effective ASFV vaccine stems from the virus’s sophisticated immune evasion mechanisms and antigenic complexity. This review argues that a safe and efficacious solution lies in a paradigm shift towards rational vaccine design centered on defined T-cell epitopes. Overcoming the bottlenecks in epitope identification—through elucidating SLA presentation rules, improving predictive algorithms, and standardizing validation pipelines—is paramount. The convergence of multi-epitope nanoparticle platforms and mRNA-LNP technology offers a promising path forward. Ultimately, unraveling the intricacies of ASFV-specific T-cell immunity and leveraging these next-generation modalities represent our most viable strategy to finally curb the devastating global impact of African swine fever.

## Figures and Tables

**Table 1:** Summary of major ASFV vaccine platforms.

Vaccine Platform	Antigen Structure	Efficacy/Protection	Biosafety Concerns
Inactivated	Whole virion, chemically or physically inactivated.	Poor to none. Fails to induce protective immunity even with modern adjuvants.	Safe but poor immunogenicity.
Subunit	Single or cocktail of recombinant structural proteins.	Inadequate. Partial reduction in viral load may occur, but fails to prevent clinical disease.	Generally safe. Limited immunogenicity. Potential for poor protective responses.
Natural attenuated	Genetically undefined attenuated virus.	Efficacy depends on dose, route, and host factors.	Residual virulence (fever, lesions, abortion). Potential for persistent infection and transmission.
Gene knockout attenuated	Virus with deletion of specific virulence genes.	Often strong homologous protection. Cross-protection is frequently inadequate.	Residual virulence, risk of reversion to virulence, and potential emergence of new strains.

**Table 3:** Comparison of widely used T-cell epitope prediction tools for swine.

Tool	Principle and Algorithm	Advantages	Limitations for ASFV/SLA
IEDB (IEDB.org, access on 6 June 2025)	Platform aggregating multiple methods (NetMHCpan, SMM, etc.).	Most widely used; provides consolidated results from various algorithms; includes 3D structural data.	Predictions are primarily based on HLA data; results from different algorithms can be inconsistent.
NetMHCpan (services.healthtech.dtu.dk, access on 6 June 2025)	Artificial neural network trained on MS-eluted peptide data.	Broad coverage of MHC-I alleles (including SLA); high accuracy for HLA; continuously updated.	Performance for SLA is still lower than for HLA due to less training data; limited number of SLA alleles covered.
MixMHCpred (mixmhc2pred.gfellerlab.org, access on 6 June 2025)	Motif deconvolution algorithm trained on MS-eluted data.	Performs well for alleles with similar binding motifs.	Limited number of SLA alleles are currently supported.
AlphaFold 2/3 (alphafoldserver.com, access on 6 June 2025)	Deep learning for predicting protein–peptide complex structures.	Provides structural insights into pSLA binding; not limited by allele-specific training data.	Predicts structure but not binding affinity score directly; requires expert interpretation.

## Data Availability

No new data were created or analyzed in this study.
